# Milk Fatty Acid Profile and Production Traits in Lithuanian Local and Holstein Cattle Breeds

**DOI:** 10.3390/ani16071083

**Published:** 2026-04-01

**Authors:** Ramutė Mišeikienė, Saulius Tušas, Elena Bartkienė, Jolita Šarkauskienė, Paulius Matusevičius

**Affiliations:** 1 Institute of Animal Rearing Technologies, Veterinary Academy, Lithuanian University of Health Sciences, Tilžės 18, LT-47181 Kaunas, Lithuaniaelena.bartkiene@lsmu.lt (E.B.);; 2 Department of Animal Nutrition, Veterinary Academy, Lithuanian University of Health Sciences, Tilžės 18, LT-47181 Kaunas, Lithuania

**Keywords:** cow, fatty acids, local breed, milk, parity, production

## Abstract

White-backed and Ash-grey cows yielded an average of 6212 kg and 6078 kg of milk per year, with 4.25–4.28% fat and 3.37–3.40% protein, while Holsteins averaged 10,694 kg with 4.47% fat and 3.45% protein. The study aimed to analyse the fatty acid (FA) profile of milk from two local cow breeds, compare them with that of Holstein cows, and evaluate the influence of lactation number, productivity indicators and milk colour. The study was carried out with White-backed (n = 40), Ash-grey (n = 49), and Holstein (n = 51) cows. Based on lactation number, cows were divided into two groups. Composite milk samples from all quarters of each cow were collected. Two additional milk samples were taken: 1st to assess milk colour and the second to determine the fatty acids profile. Productivity data of cows were provided from the Livestock Information System. Statistical analysis included calculating means and standard deviations or standard errors of the mean. One-way and two-way ANOVA (breed and lactation) were used, and Duncan’s test was applied to compare mean values between groups. White-backed cows contained greater proportions of C17:0, C17:1, C18:3 ω3, and C20:0 (*p* < 0.05), as well as higher total omega-3 and polyunsaturated fatty acid (PUFA) contents. Parity did not have a significant effect on productivity indicators, but parity influenced the FA profile. Milk from 1st-lactation Ash-grey cows showed higher levels of PUFA and omega-6 fatty acids (*p* < 0.05). Milk from Ash-grey cows has more favourable visual attributes, indicating improved suitability for dairy processing, where colour uniformity is important for technological quality.

## Simple Summary

Local dairy breeds are generally better adapted to local climatic and feeding conditions and often exhibit greater disease resistance and longevity. Milk from local cow breeds may have unique quality traits, including a favourable fatty acid profile, which enhances overall milk quality and increases the nutritional value and health benefits of dairy products. The study was carried out with White-backed, Ash-grey and Holstein cows. On average, White-backed and Ash-grey cows produced 6212 kg and 6078 kg of milk per year, with fat contents of 4.25% and 4.28%, protein contents of 3.37% and 3.40%, respectively, while Holsteins averaged 10,694 kg of milk with 4.47% fat and 3.45% protein. For the analysis of milk fatty acid profile, productivity parameters and colour, a total of 1120 milk samples were taken. Milk from White-backed cows contained greater proportions of long-chain fatty acids (C17:0, C17:1, C18:3 ω3, C20:0) (*p* < 0.05), as well as higher total omega-3 and polyunsaturated fatty acid (PUFA) contents. Parity did not have a significant effect on productivity indicators in the studied breeds; however, lactation number influenced milk fatty acid profile. Milk from 1st lactation Ash-grey cows showed significantly higher levels of PUFA and omega-6 fatty acids, suggesting that parity contributes to variations in the concentration of biologically valuable fatty acids in milk. Assessment of milk colour parameters demonstrated that milk from Ash-grey cows possessed more favourable visual attributes, suggesting improved suitability for dairy processing, where colour uniformity is important for technological quality.

## 1. Introduction

Local cattle breeds are a vital genetic resource that has contributed to the biodiversity, adaptation to local environmental conditions, and sustainability of the livestock system [1]. Several traditional breeds are distinguished in Lithuania, which, although less common than commercial high-yield breeds, are characterised by valuable characteristics, such as disease resistance, longevity and the ability to use local feed effectively. The White-backed and Ash-grey cattle breeds are linked to the history of Lithuania and have great cultural value. The White-backed cattle breed has been known in Lithuania since ancient times, and at the beginning of the 20th century, it accounted for 10% of all cattle. Meanwhile, Lithuanian Ash-grey cattle in the 16th century accounted for about 6% of all mentioned cattle [2]. Cattle of these two breeds are kept mostly on private farms. Both breeds maintain their distinctive traits and are appreciated for their adaptability, good health, and calm temperament. As of 30 September 2025, records in the Livestock Information System showed 490 local Ash-grey and 384 local White-backed dairy cattle were recorded. On average, the productivity of White-backed cows was 6212 kg of milk per year, with fat and protein percentages of 4.25% and 3.37%, respectively. During the year, Ash-grey on average produced 6078 kg of milk with a fat content of 4.28% and a protein content of 3.40%. The average productivity of Holsteins was 10,694 kg of milk, with a fat percentage of 4.47% and a protein percentage of 3.45%. The average number of lactations for White-backed cows was 3.3, Ash-grey cows was 3.7, and Holsteins was 2.0 lactations [3].

The genetic uniqueness and long-term selection of these breeds give them a distinctiveness that is important not only for animal welfare but also for milk quality. Growing attention is given not only to productivity but also to milk quality, especially fatty acid composition, which affects human health, the technological quality of raw milk, and the sensory quality of dairy products [4].

Dairy fat is one of the most complex natural fats because of its fatty acid (FA) composition, and it varies much more than other milk components (e.g., protein, lactose) in concentration and composition [5]. High variability was found not only in fat content, but also in the proportion of FA and their groups [6]. To evaluate the nutritional quality of bovine milk fat, it is essential to analyse the overall FA profile of milk. Ruminant milk fat contains more than 400 FA [7]. The physicochemical properties, sensory attributes, and nutritional quality of milk and dairy fat are largely influenced by their FA composition [8]. Most milk fat consists of saturated fatty acids (SFA), which account for approximately 70%, while UFA (unsaturated fatty acids) represent around 30% [9].

In dairy farming, the FA profile plays a decisive role in determining the technological quality of raw milk [10]. This can significantly affect the production of value-added dairy products; therefore, FA has great economic importance [4]. Most SFAs are synthesised de novo (in mammary epithelial cells), whereas most UFAs are obtained from the diet and body fat stores [11]. Milk fat content depends on many factors, such as feeding method, age of cows, parity, which are valuable sources of SFA, monounsaturated fatty acids (MUFA) and polyunsaturated fatty acids (PUFA) [5,12,13], breed, parity and lactation stage, and nutrition [14] but according to Młynek et al. [15], diet did not directly influence the FA profile in the milk. Samková et al. [16] state that cattle breed can have a significant impact on the FA profile of milk, but Hanuš et al. [4] opinion is the opposite. He indicates that the influence of breed is limited. Several factors influence milk colour, including the genetics and breed of the animals [17], their diet, stage of lactation, parity, milking time, udder health status, and season [18,19].

There are limited worldwide studies analysing the FA profile of milk from local cattle breeds and comparing it with that of Holstein and other breeds, as noted by Hanuš et al. [4]. According to him, there is insufficient research on the fatty acid profiles of milk from different breeds under the same nutritional conditions, for example, comparing Holsteins with local extensive breeds. Therefore, our study aimed to analyse the FA profile of milk from Lithuanian local cattle breeds, compare it with that of Holstein cows, and evaluate the effect of lactation number. We also assessed and compared productivity indicators and milk colour characteristics among the studied breeds.

## 2. Materials and Methods

### 2.1. Animals and Sampling

The study was carried out with 140 lactating cows, including White-backed (n = 40), Ash-grey (n = 49), and Holstein (n = 51). The cows were kept tied, with straw bedding. The cows were divided into two groups based on lactation number: Group I—cows in their 1st lactation; and Group II—cows in their 2nd lactation and older. All cows were kept under highly similar housing conditions and were fed a complete diet (haylage 22–25 kg, hay 8–10 kg, and concentrates 4–6 kg (extensive farming)/day) that met their physiological needs. All animals were kept according to the established hygiene requirements.

The research was conducted on 8 private dairy farms. Cows were milked using a pipeline milking system. Milk samples were collected during the control milking from the pipeline using a milk metre. They were obtained by individually taking milk samples and measuring total milk yield. A composite milk sample from all four teats of each analysed cow was collected into a sterile tube and delivered to the laboratory for milk composition analysis. Two additional milk samples were taken: 1st to assess milk colour and the second to determine the fatty acids profile. Productivity data of cows were provided from the Livestock Information System. The research was conducted from January to April 2025. During this period, a total of 1120 milk samples were taken.

The research was performed in accordance with the provisions of the Law on Animal Welfare and Protection of the Republic of Lithuania, No XI-2271 [20].

### 2.2. Ethics Declaration

The study was conducted according to the methodology outlined in the Law on the welfare of the farm animals of the Republic of Lithuania and complied with Directive 2010/63/EU of the European Parliament and the Council on the Protection of Animals used for scientific purposes. Ethical approval was obtained for the study (Ethics approval No. 2025-BEC3-T-047).

### 2.3. Analysis of Milk Productivity Indicators

Fat, protein and lactose content were determined by the LactoScope FTIR infrared instrument (Perten Instruments, Stockholm, Sweden). Milk analysis was performed in the Joint Stock Company Pieno Tyrimai accredited central milk testing laboratory (Kaunas, Lithuania).

### 2.4. Analysis of FA Profile

The complete lipids from untreated milk were extracted using the Folch method [21] with a mixture of chloroform and methanol (in a 2:1, *v*/*v* ratio). After centrifugation, the organic layer was separated and transferred to a glass vial. Subsequently, it was rinsed with a 0.9% sodium chloride (NaCl) solution in water. The organic phase was then dried by evaporating it to dryness using a stream of nitrogen gas. The dried lipid residue is dissolved in hexane, and then it is derivatised using a 0.2 M potassium hydroxide (KOH) solution in methanol. The fatty acid (FA) composition of the milk samples was determined using a GC-MS-QP2010 (Shimadzu, Kyoto, Japan) gas chromatograph with a mass spectrometer [22]. The separation process was carried out using a Stabilwax-MS column with dimensions of 30 m in length, 0.25 mm internal diameter (ID), and a 0.25 µm film thickness (df). This column was supplied by Restek Corporation, located in Bellefonte, PA, USA. Fatty acid methyl esters (FAME) concentrations were determined using a calibration curve, and results were expressed as a percentage of the total FAME concentration in the sample.

### 2.5. Milk Colour Evaluation

The colour of the milk was evaluated using a Minolta Chroma Metre (CR-400; Minolta, Osaka, Japan) colourimeter. A volume of 10 mL of each milk sample was measured on a plate, and the results were expressed using the CIE L*a*b* colour space. In this system, lightness (L*) ranges from 0 (black) to 100 (white); the a* value represents the green–red axis (−a* = green, +a* = red), and the b* value represents the blue–yellow axis (−b* = blue, +b* = yellow). Higher absolute values of a* or b* indicate stronger colour intensity, while values closer to zero correspond to less saturated colours. This colour space is commonly used in colourimetric analysis of food samples [23].

### 2.6. Statistical Analysis

The statistical analysis included the calculation of arithmetic means and standard deviations (SD) or standard errors of the mean (SEM). Data were analysed using two-way ANOVA (considering breed and lactation) and one-way ANOVA (considering either breed or lactation). Differences in mean values between breed groups were assessed using Duncan’s test. All calculations were performed with Statistica 2019 software, and significance was set at *p* ≤ 0.05.

## 3. Results

### 3.1. Productivity Parameters and Parity

The primary objective of this study was to compare the milk composition indicators and fatty acid profile of Lithuanian local (White-backed and Ash-grey) and Holstein dairy cow breeds.

The analysis revealed that Holstein cows had the highest milk yield (Table 1). The Holstein produced on average 12.05 kg and 12 kg more milk than the Ash-grey and White-backed cows, respectively. The highest protein percentage was determined in Holstein milk, while the lowest was in the White-backed cow breed. Although breed did not have a statistically significant effect on fat and lactose concentrations, milk from White-backed cows had the highest fat content, which was 0.25% higher than that of milk from Holstein cows.

Lactation number did not affect milk yield, fat, protein and lactose concentration (Table 2).

### 3.2. Fatty Acids Profile

The results for breed differences in the fatty acid profile are presented in Table 3. Breed had a significant effect on C10:0, C12:0, C13:0, C14:0, C15:0, C17:0, C17:1, C18:0, C18:3 ω3, C20:0, C20:4 ω6 and omega-3 acids. The main differences were detected between Holstein and local cow breeds. Specifically, milk of Holsteins had 0.82% more C10:0 than Ash-grey milk and 0.78% more than White-backed milk. Additionally, Holstein milk had 1.01% more C12:0 than White-backed and 0.98% more than Ash-grey milk. Holstein milk contained 0.1% more C13:0 than both local breeds. Furthermore, the content of C14:0 in Holstein milk was 1.15% and 1% higher than in the milk of White-backed and Ash-grey local breeds, respectively. The highest level of C15:0 was found in the milk of White-backed cows (2.00), and the lowest in Ash-grey milk (1.60). Arachidonic C20:4 ω6 in Holstein cows’ milk was 0.05% higher than in White-backed milk (*p* = 0.012). The milk of Holstein cows, compared with that of two local breeds, had the lowest content of C17:0, C17:1, C18:3 ω3, C20:0, and omega-3 (*p* < 0.05). Milk from Holstein cows contained lower percentages of several fatty acids compared to milk from White-Backed cows. Specifically, the levels of C17:0, C17:1, C18:3 ω3, C20:0, and omega-3 in Holstein milk were lower by 0.35%, 0.13%, 0.62%, 0.08%, and 0.62%, respectively, than those in White-Backed milk. In Ash-grey milk, the percentages of these fatty acids were also lower by 0.3%, 0.12%, 0.37%, 0.01%, and 0.37%. These differences were statistically significant (*p* < 0.05). Meanwhile, the highest percentage of C18:0 was fixed in the Ash-grey cows’ milk. It was 2.51% and 1.21% higher than in Holstein and Ash-grey milk (*p* = 0.010).

When comparing the proportions of the main FA groups (SFA, MUFA, PUFA) in the milk of local breeds, such as White-backed and Ash-grey cows, with those of Holstein cattle, slight but non-significant differences were observed. Holstein cow milk contains the highest proportion of saturated fat, with levels 2.3% and 3.52% higher than those found in White-backed and Ash-grey breeds, respectively. Ash-grey cows produced milk with the highest MUFA percentage, which was 3.4% greater than that of Holstein cows. The highest concentration of PUFA was observed in the milk of White-backed cows, exceeding that of Ash-grey and Holstein cows by 0.12% and 0.25%, respectively. The highest concentration of omega-3 was determined in White-backed cows‘ milk (*p* = 0.000).

The effect of breed on the fatty acid profile by lactation number is presented in Table 4. Comparison of individual fatty acid concentrations according to cow lactation groups revealed that the concentration of certain SFA—butyric acid (C4:0), lauric acid (C12:0), myristic acid (C14:0), and arachidic acid (C20:0)—was significantly higher in cows of the 2nd lactation group (*p* < 0.05). In contrast, the concentrations of linoleic acid (C18:2), dihomogammalinolenic acid (C20:3 ω-6), and arachidonic acid (C20:4 ω-6) were higher in 1st lactation cows’ milk (*p* < 0.05).

The influence of lactation on the fatty acid profile differed between breeds. The highest concentration of C4:0 fatty acid was observed in the milk of Ash-grey cows in their 2nd lactation group (*p* = 0.092). In contrast, milk from 1st lactation Ash-grey cows had significantly higher levels of C18:2 ω6, C20:3 ω6, and C20:4 ω6 compared with the other breeds (*p* < 0.05). Second-lactation group Holstein cows exhibited the highest C14:0 content (*p* = 0.042), along with elevated levels of C6:0 and C16:0 relative to the other two breeds. Milk from 1st lactation White-backed cows was characterised by the highest C18:3 ω3 content (*p* = 0.007). Furthermore, milk from 1st lactation Ash-grey cows was characterised by significantly higher proportions of PUFA and omega-6 fatty acids (*p* < 0.05). In contrast, while not reaching statistical significance, the highest SFA levels were observed in the 2nd lactation group of Holstein, and the highest MUFA levels in the 1st lactation Ash-grey cows.

### 3.3. Milk Colour Coordinates

**Figure 1:**
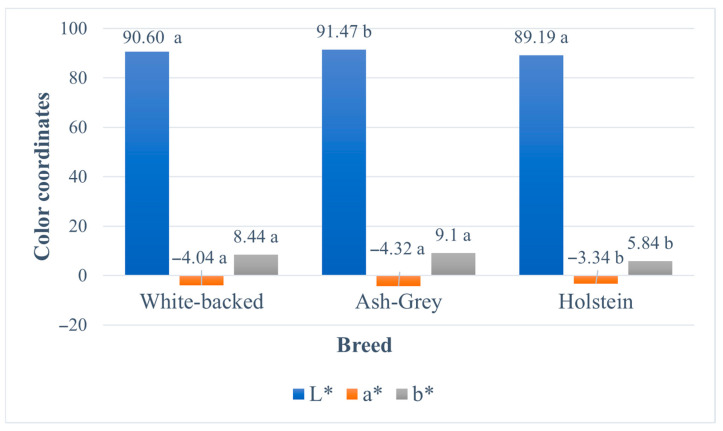
Milk colour coordinates according to cow breed. L* indicates lightness; a* represents the red-green axis; and b* represents the yellow-blue axis. Means in the same column followed by different letters (a, b) are significantly different according to the Fisher LSD criterion test (*p* ≤ 0.05).

Figure 1 presents the findings from the milk colour analysis of the studied cow breeds. Because the milk colour of both lactation groups (1st and 2nd) was nearly identical, the results are presented jointly. The colour comparison was conducted by calculating the mean of the chromatic components (L*, a*, and b*). Milk from Ash-grey cows was found to be statistically the lightest (L*) compared with that from White-backed and Holstein cows (*p* = 0.044). Milk samples from all examined breeds showed negative a* values, indicating a greenish colouration. The most intense greenish hue was observed in Ash-grey cow milk, followed by White-backed, and the least pronounced greenish colouration was determined in Holstein milk (*p* = 0.000). In addition, Ash-grey cows produced significantly yellower milk, with a b* value of 0.66 and 3.26 higher than White-backed and Holsteins (*p* = 0.030).

## 4. Discussion

### 4.1. Cow Breed, Productivity, Parity

Milk composition, including its fat, protein, and lactose content, is influenced by both genetic and non-genetic factors, such as breed, age, lactation stage, animal health, feeding, season, milking technique, number of lactations, individual cows, and environmental factors [24,25]. Palii et al. [26] note that each breed has specific traits that are influenced by heredity and long-term selection processes.

High-producing breeds like Holstein cows have been selectively bred for milk yield, leading to superior production but often lower fat content. Conversely, local breeds have not been deliberately improved, which is why their productivity is lower; however, they are better adapted to their environments, resulting in higher fat and protein levels and more consistent production under changing conditions.

The results of this study are consistent with well-established patterns. We observed that the protein content in Holstein milk was significantly higher compared to the two local breeds analysed (*p* = 0.000), but the fat percentage in Holstein milk was the lowest. Similar breed-related differences have been reported in previous studies. For example, Johansson [27] found that Swedish Mountain Cattle (SMC) produced milk with higher lactose but lower fat and protein content than Swedish Holstein cattle. Other studies have shown higher milk yield in Holstein cows compared to the local Czech Fleckvich breed (*p* < 0.05) [4]. Maurice-Van Eijndhoven et al. [28] compared the milk production characteristics of Holstein-Friesian (HF), Meuse-Rhine-Yssel (MRY), Dutch Friesian (DF), and Groningen White Headed (GWH) cows. They found that Holstein cows exhibited the highest average daily milk yield at 28.5 kg, whereas GWH cows had the lowest yield at 24 kg. GWH cows produced milk with a lower fat content and the lowest protein content, while higher values were noted in MRY and DF breeds. Meanwhile, studies conducted in Iran [29] showed that the milk of local Sarabi cows was not only higher in fat but also contained more protein compared to Holsteins (*p* = 0.0340). The highest lactose content was observed in White-backed and Holstein milk (*p* > 0.005). Similar results were obtained in the study by Johansson et al. [27]. They found the highest lactose content in SMC and Swedish Holstein cattle.

The differences among Holstein and local breeds can be explained not only by genetic potential but also by genotype-environment interaction, which influences how animals express their production traits under specific management and environmental conditions. Local breeds are genetically more resilient and better adapted to low-input systems, whereas high-producing breeds tend to perform optimally under intensive management.

In addition to breed effects, parity is an important factor that influences milk productivity and composition [30]. Kramarenko et al. [31] note that lactation number has a significant effect on all milk yield traits. Research by Wathes et al. [32] showed that milk yield generally increases from the 1st to the 3rd or 4th lactation, after which it gradually declines. Cows in their 1st lactation typically produce less milk as they are still growing. Our results partly support this trend, as milk yield in White-backed cows was higher in the 2nd lactation group compared to the 1st lactation cows (*p* > 0.05). In contrast, older lactations may see a decline in productivity due to age and health-related issues [17]. Variations in milk composition across lactations have also been reported, with some studies indicating higher concentrations of fat and protein in either early or later lactations depending on physiological and management factors [33,34]. Our findings have shown that protein content in all analysed breeds was higher in the 2nd lactation group, but fat content in the 1st lactation Holstein milk was higher. However, the extent of these changes may depend on the genetic potential of the breed. Highly productive breeds, such as Holsteins, tend to show more variation in productivity across lactations, while indigenous breeds generally maintain more stable productivity levels [35]. In our research, the number of lactations did not have a significant effect on productivity traits, suggesting that breed-related genetic factors may play a more dominant role than parity under the studied conditions.

### 4.2. Cow Breed and FA Profile

One of the primary factors influencing the fatty acid composition of milk is the breed of cattle [36]. We found limited studies analysing the fatty acid profiles of local and commercial cattle breeds, so we focused on similar research conducted in Poland [37], the Czech Republic [38], the Netherlands [28,39], Ukraine [40] and Iran [29]. The results from these studies were a basis for comparison with our local cattle breeds and Holstein cows.

Analysis of individual fatty acids revealed notable differences between Holstein and the local cow breeds. Holstein milk contained significantly higher concentrations of SFA, including lauric and myristic acids, capric, tridecylic, while it exhibited significantly lower levels of stearic and arachidic acids. White-backed cows’ milk showed significantly higher concentrations of margaric, cis-10-heptadecenoic and α-linolenic acids, and significantly lower concentrations of arachidonic acid. Meanwhile, Ash-grey milk contained the lowest content of pentadecylis acid (*p* = 0.001).

Similar trends have been noted in other studies. In a study by Adamska et al. [37], the Polish Red and White breeds exhibited relatively lower nutritionally significant amounts of C14:0 and C16:0 fatty acids compared to other breeds. Local cattle produced milk with a nutritionally superior fat composition compared to higher-yielding breeds. However, local cows are often kept in extensive farming conditions. Our findings showed that the contents of myristic and palmitic acids in both local breeds were lower than those in Holstein milk. Maurice-Van Eijndhoven et al. [28] also determined breed-specific differences in milk fatty acids among HF, MRY, DF, and GWH cows. Short- and medium-chain saturated fatty acids were relatively similar across local breeds. However, GWH tended to have slightly lower concentrations, and Holstein values were intermediate. In contrast, some long-chain fatty acids exhibited greater variation among breeds, with Holsteins typically having higher concentrations. The SFA proportion, which is generally considered unfavourable to human health, was lower in the GWH breed. The proportions of conjugated linoleic acid and the unsaturation index, which are positively associated with human health, were both highest in GWH cows [39].

Analysing the fatty acid profile by fatty acid groups, Wang et al. [41] reported that SFA are the most abundant fatty acids in milk. According to Månsson [9], the proportion of SFA in cow milk ranges from 67.1% to 74.4%. A study conducted by Ukrainian scientists [40] evaluated the FA composition of milk from the indigenous Ukrainian Grey and Ukrainian White-headed breeds. The researchers compared these indigenous breeds with commercial dairy cattle populations, including Holsteinized Ukrainian Black and White cows and purebred Holsteins. The results showed that although the total SFA content in Holsteinized Ukrainian Black and White cow milk is similar, the level of essential α-linolenic acid is about 10 times lower than in two indigenous breeds (by 66.61% and 68.37%). Additionally, Holsteinized milk contains higher levels of some less desirable SFA—caproic, caprylic, lauric, and myristic acids—compared to indigenous Ukrainian breeds. In contrast, our study found higher SFA content in both local breeds compared with Ukrainian local breeds. The highest SFA content was observed in Holstein milk, although the differences were not statistically significant. Research in the Czech Republic also revealed that milk fat from Czech Pied cattle contains significantly fewer SFA compared to Holstein cows [38]. Bassiri et al. [29] analysed the indigenous Sarabi and Holstein milk FA profiles and observed that both breeds exhibited higher concentrations of SFA and long-chain fatty acids, along with slightly lower MUFA levels, compared to Holstein cows (*p* > 0.05). As no studies were found specifically examining whether fatty acid profiles differ between local and Holstein cows according to lactation number, the further analysis was based on available literature describing the general effect of lactation number on milk fatty acid composition, as well as on the results obtained in our study. According to Frizari et al. [13], multiparous cows produce milk richer in saturated and short-chain fatty acids, but lower in unsaturated, medium-, and long-chain fatty acids. In contrast, first-lactation cows have higher PUFA, MUFA, and omega-6 levels, while later-lactation cows show higher SFA, a pattern consistent across breeds.

With respect to parity, in the study of Rodríguez-Bermúdez et al. [36], C18:1 and MUFA were higher in primiparous cows, while C16:0, SFA and SCFA tended to be higher in multiparous cows. These findings are in accordance with studies, where 1st parity cows had relatively higher proportions of UFA and oleic acids and lower amounts of SFA and C16:0 in milk fat than later-parity cows [42]. The mammary gland of 1st parity cows was metabolically less active, having a lower expression of FA synthase, than in later parity cows, and this could explain the lower proportions of de novo FA [42]. Rodríguez-Bermúdez et al. [36] noted second-parity cows produced more C16:0, C18:0, total SFA, and SCFA (C2:0–C6:0) than primiparous cows. Our results indicated that milk from Ash-grey cows in their 2nd and later lactations contained significantly higher levels of butyric (0.29%) and arachidic (0.03%) compared to primiparous cows. Conversely, primiparous Ash-grey cows produced milk with higher concentrations of linoleic, dohomogamalinoenic fatty acids. In White-backed cows, the 2nd lactation group had 0.71% higher lauric and 0.05% lower alpha-linolenic acid compared to 1st lactation cows. Milk of Holstein cows in the 2nd lactation group contained 1.37% more myristic acid. The significantly higher concentrations of PUFA (0.57%, *p* = 0.010) and omega-6 fatty acids (0.56%, *p* = 0.007) were observed in milk from 1st lactation Ash-grey cows. Morales-Almaráz et al. [43] observed higher proportions of MUFA and lower proportions of SFA in 1st and 2nd parity cows. Our research has shown that in the 1st lactation, Holstein cows‘ milk SFA proportion was higher, and MUFA was lower.

The observed differences in milk FA composition between Holstein and local White-backed and Ash-grey breeds indicate that breed influences specific FA, particularly omega-3 and minor SFA. Holstein milk had the lowest levels of C17:0, C17:1, C18:3 ω3, C20:0, and total omega-3, likely reflecting the genetic selection for high milk yield, which is often associated with a dilution of certain beneficial FA. In contrast, White-backed milk contained the highest concentrations of omega-3 and PUFA, suggesting that local breeds may offer advantages in milk quality and functional FA content despite lower total milk yield. Ash-grey milk exhibited intermediate levels, with the highest C18:0 percentage, highlighting breed-specific patterns in SFA. Meanwhile, the proportions of SFA, MUFA, and PUFA differed only slightly, indicating that overall milk fat composition remains largely comparable across breeds. Lactation influenced specific FA in a breed-dependent manner: 2nd lactation group cows had higher contents of certain SFA, whereas 1st lactation cows had elevated linoleic and omega-6 FA. However, parity did not significantly affect productivity indicators, suggesting that breed genetics exerts a stronger influence on milk FA composition. Overall, these results demonstrate that breed-related differences in milk are most pronounced at the level of individual FA. This highlights the potential of local Lithuanian breeds to produce milk with enhanced nutritional properties, which could be considered in breeding and dairy management strategies.

### 4.3. Milk Colour

Milk colour is associated with cow breed, parity, stage of lactation, udder health status, milk and fat yields, and genetic traits [19,44]. Milk colour parameters can serve as a non-invasive indicator to assess the productive and health status of dairy cows, as well as to determine the quality of the produced milk [45]. Scientists reported that milk colour varies according to lactation number. According to Adyan et al. [45], cows in their 1st parity and the early lactation stage exhibit the highest values of lightness (L*) and yellowness (b*). The yellowish tones in milk are primarily due to β-carotene levels that are affected by cattle breed and dietary carotenoid intake, while riboflavin (vitamin B_2_) in whey imparts subtle greenish hues, particularly evident in skim milk [46]. Our findings showed that the milk of all breeds, regardless of lactation group, was very light, had a greenish tint and was characterised by a noticeable, but not weak, yellowness. Milk from 1st lactation cows was slightly lighter; however, this difference was not visually noticeable. Meanwhile, Milovanovic et al. [23] noted that parity significantly determined L* and b* colour parameters. Primiparous cows exhibited the highest L* values and b* values compared with multiparous cows. Findings of Milovanovic et al. [23] show that higher fat and total solids in multiparous milk are associated with changes in fat-soluble pigments, affecting lightness and yellowness. In contrast, the lower fat and solids content of primiparous milk is reflected in its lighter and more yellow appearance, whereas multiparous milk tends to be darker with less pronounced yellow hues. But our results are opposite: milk from the 2nd lactation cows had a slightly more pronounced greenish and yellow tint, although the differences between lactations were small. Milk from local breeds was greener than that of Holsteins, consistent with Milovanovic et al. [23], who reported that milk from Norwegian Red and Montbéliarde cows was greener than Holstein milk. The milk colour was compared among breeds, revealing that Ash-grey cows had a significantly higher mean lightness (L*) than White-backed and Holstein cows. Milk from Holstein cows exhibited the lowest intensity of yellow (b*) and green (a*) colouration (*p* < 0.05). Although White-backed cows produced milk with the highest fat content, the most intensely yellow (b*) milk came from Ash-grey cows.

## 5. Conclusions

Although Holstein cows demonstrated the highest milk yield and protein content, the local Ash-grey and White-backed breeds showed favourable milk composition traits, underscoring the importance of preserving local dairy cattle breeds.

Statistically significant differences in milk fatty acid composition were identified between Holstein and Lithuanian local breeds (White-backed and Ash-grey). Holstein milk was characterised by higher concentrations of medium-chain saturated fatty acids (C10:0, C12:0, C13:0, C14:0). In contrast, milk from White-backed cows contained greater proportions of long-chain fatty acids (C17:0, C17:1, C18:3 ω3, C20:0) as well as higher total omega-3 and polyunsaturated fatty acid contents. Nevertheless, a general tendency was observed for Holstein milk to contain relatively more saturated fatty acids, while milk from local breeds—particularly White-backed cows—showed a more favourable profile in terms of omega-3 and polyunsaturated fatty acids.

Parity did not have a significant effect on productivity indicators in the studied breeds; however, lactation number influenced milk fatty acid composition. Milk from 1st-lactation Ash-grey cows showed higher levels of PUFA and omega-6 fatty acids (*p* < 0.05), suggesting that parity influences the concentration of biologically valuable fatty acids in milk. In general, differences between breeds were most evident at the level of individual fatty acids, highlighting the potential of local Lithuanian breeds to produce milk with enhanced nutritional value, which could be considered in breeding and dairy management strategies.

Furthermore, assessment of milk colour parameters demonstrated that milk from Ash-grey cows has more favourable visual attributes, suggesting improved suitability for dairy processing, where colour uniformity is important for technological performance and consumer acceptance.

## Figures and Tables

**Table 1:** Effect of cow breed on average productivity indicators per day.

Parameters	Breed			SEM	*p*-Value
	White-Backed	Ash-Grey	Holstein		
Milk yield, kg	17.92 ^a^	17.96 ^a^	29.97 ^b^	0.75	0.000
Fat, %	4.20	4.10	3.95	0.09	0.756
Protein, %	3.12 ^a^	3.44 ^b^	3.61 ^b^	0.05	0.001
Lactose, %	4.41	4.36	4.41	0.03	0.685

Means in the same row followed by different inline letters ^(a, b)^ are significantly different according to the Fisher LSD criterion test (*p* ≤ 0.05).

**Table 2:** Productivity indicators between cow breeds depending on lactation number.

Parameters	Breed	Lactation Group		SEM	*p*-Value
		I	II		
Milk yield, kg	White-backed	17.10	18.73	1.03	0.497
	Ash-grey	18.71	17.20	0.93	0.451
	Holstein	31.49	28.45	2.54	0.588
Fat, %	White-backed	4.09	4.31	0.13	0.448
	Ash-grey	3.86	4.34	0.15	0.136
	Holstein	4.27	3.63	0.24	0.216
Protein, %	White-backed	3.11	3.12	0.07	0.972
	Ash-grey	3.38	3.50	0.06	0.319
	Holstein	3.52	3.70	0.13	0.557
Lactose, %	White-backed	4.49	4.32	0.04	0.072
	Ash-grey	4.38	4.33	0.04	0.563
	Holstein	4.37	4.45	0.04	0.404

Values are expressed as mean value ± SEM.

**Table 3:** Influence of cow breed on FA composition (% of total fats).

Fatty Acid	Breed			SEM	*p*-Value
	White-Backed	Ash-Grey	Holstein		
Butyric C4:0	2.85	2.90	2.66	0.05	0.523
Caproic C6:0	1.94	1.92	1.99	0.03	0.798
Caprylic C8:0	1.20	1.21	1.39	0.03	0.152
Capric C10:0	2.92 ^a^	2.88 ^a^	3.70 ^b^	0.08	0.007
Lauric C12:0	3.61 ^a^	3.64 ^a^	4.62 ^b^	0.10	0.012
Tridecylic C13:0	0.08 ^a^	0.08 ^a^	0.18 ^b^	0.01	0.000
Myristic C14:0	12.29 ^a^	12.44 ^a^	13.44 ^b^	0.16	0.042
Myristoleic C14:1	1.26	1.40	1.39	0.05	0.361
Pentadecylic C15:0	2.00 ^a^	1.60 ^b^	1.91 ^a^	0.05	0.001
Palmitic C16:0	34.94	33.05	36.00	0.52	0.150
Palmitoleic C16:1	2.46	2.23	2.08	0.58	0.086
Margaric C17:0	1.01 ^a^	0.71 ^b^	0.66 ^b^	0.03	0.001
cis-10-Heptadecenoic C17:1	0.48 ^a^	0.36 ^b^	0.35 ^b^	0.02	0.005
Stearic C18:0	9.37 ^a^	10.58 ^a^	8.07 ^b^	0.27	0.010
Oleic C18:1	19.82	21.38	18.15	0.43	0.063
Linoleic C18:2 ω6	2.14	2.34	2.45	0.07	0.334
α-Linolenic C18:3 ω3	1.14 ^a^	0.77 ^b^	0.52 ^b^	0.04	0.001
Arachidic C20:0	0.20 ^a^	0.19 ^a^	0.12 ^b^	0.01	0.002
Dohomogamalinoenic C20:3 ω6	0.10	0.12	0.11	0.01	0.126
Arachidonic C20:4 ω6	0.12 ^a^	0.16 ^b^	0.17 ^b^	0.01	0.012
C22:0	0.13	0.11	0.07	0.001	0.096
FA groups					
Saturated fatty acids (SFA)	72.50	71.28	74.80	0.47	0.099
Monounsaturated fatty acids (MUFA)	24.01	25.36	21.96	0.43	0.061
Polyunsaturated fatty acids (PUFA)	3.49	3.37	3.24	0.10	0.722
Omega 6	2.36	2.60	2.72	0.07	0.243
omega-3	1.14 ^a^	0.77 ^b^	0.52 ^b^	0.04	0.000

Values are expressed as mean value ± SEM; Means in the same row followed by different inline letters ^(a, b)^ are significantly different according to the Fisher LSD criterion test (*p* ≤ 0.05).

**Table 4:** Effect of breed and lactation on milk fatty acid composition (%).

Fatty Acid	Breed	Lactation Group		SEM	*p*-Value
		1	2		
Butyric C4:0	White-backed	3.01	2.68	0.09	0.092
	Ash-grey	2.75	3.04 *	0.08	0.035
	Holstein	2.64	2.73	0.06	0.825
Caproic C6:0	White-backed	1.96	1.92	0.04	0.757
	Ash-grey	1.90	1.94	0.03	0.705
	Holstein	1.92	2.06	0.04	0.634
Caprylic C8:0	White-backed	1.14	1.26	0.04	0.249
	Ash-grey	1.21	1.21	0.05	0.987
	Holstein	1.32	1.46	0.04	0.431
Capric C10:0	White-backed	2.69	3.14	0.12	0.092
	Ash-grey	2.89	2.86	0.09	0.878
	Holstein	3.42	3.97	0.10	0.526
Lauric C12:0	White-backed	3.25	3.96*	0.16	0.048
	Ash-grey	3.73	3.54	0.15	0.526
	Holstein	4.16	5.08	0.14	0.058
Tridecylic C13:0	White-backed	0.07	0.09	0.01	0.234
	Ash-grey	0.09	0.07	0.01	0.289
	Holstein	0.14	0.21	0.03	0.202
Myristic C14:0	White-backed	11.83	12.75	0.25	0.101
	Ash-grey	12.63	12.24	0.26	0.456
	Holstein	12.75	14.12 *	0.24	0.042
Myristoleic C14:1	White-backed	1.11	1.41	0.07	0.071
	Ash-grey	1.52	1.28	0.07	0.099
	Holstein	1.24	1.54	0.07	0.438
Pentadecylic C15:0	White-backed	1.95	2.05	0.08	0.634
	Ash-grey	1.66	1.53	0.06	0.238
	Holstein	1.76	2.06	0.05	0.430
Palmitic C16:0	White-backed	36.12	33.75	1.03	0.309
	Ash-grey	32.90	33.20	0.59	0.812
	Holstein	35.75	36.24	0.91	0.808
Palmitoleic C16:1	White-backed	2.35	2.57	0.49	0.310
	Ash-grey	2.37	2.09	0.53	0.073
	Holstein	2.11	2.05	0.58	0.828
Margaric C17:0	White-backed	1.06	0.95	0.04	0.309
	Ash-grey	0.72	0.70	0.03	0.807
	Holstein	0.65	0.67	0.05	0.756
cis-10-Heptadecenoic C17:1	White-backed	0.43	0.52	0.03	0.156
	Ash-grey	0.38	0.33	0.02	0.261
	Holstein	0.35	0.35	0.02	0.957
Stearic C18:0	White-backed	9.21	9.52	0.42	0.750
	Ash-grey	9.96	11.20	0.36	0.106
	Holstein	9.06	7.08	0.58	0.999
Oleic C18:1	White-backed	20.02	19.61	0.69	0.798
	Ash-grey	21.37	21.38	0.59	0.992
	Holstein	19.40	16.89	0.99	0.235
Linoleic C18:2 ω6	White-backed	2.13	2.15	0.12	0.963
	Ash-grey	2.59 *	2.08	0.09	0.009
	Holstein	2.38	2.52	0.08	0.464
α-Linolenic C18:3 ω3	White-backed	1.16 *	1.11	0.08 *	0.007
	Ash-grey	0.77	0.76	0.03	0.095
	Holstein	0.52	0.52	0.03	0.989
Arachidic C20:0	White-backed	0.19	0.20	0.01	0.710
	Ash-grey	0.17	0.20 *	0.01	0.033
	Holstein	0.12	0.11	0.01	0.404
Dohomogamalinoenic C20:3 ω6	White-backed	0.09	0.11	0.01	0.078
	Ash-grey	0.13 *	0.10	0.01	0.037
	Holstein	0.10	0.11	0.01	0.577
Arachidonic C20:4 ω6	White-backed	0.11	0.13	0.01	0.274
	Ash-grey	0.17 *	0.14	0.01	0.034
	Holstein	0.14	0.19	0.01	0.087
Behenic C22:0	White-backed	0.12	0.13	0.01	0.611
	Ash-grey	0.11	0.11	0.01	0.691
	Holstein	0.08	0.06	0.01	0.096
FA groups					
Saturated fatty acids (SFA)	White-backed	72.60	72.40	0.81	0.910
	Ash-grey	70.71	71.84	0.64	0.412
	Holstein	73.76	75.84	1.04	0.357
Monounsaturated fatty acids (MUFA)	White-backed	23.90	24.11	0.69	0.896
	Ash-grey	25.64	25.08	0.59	0.661
	Holstein	23.10	20.82	0.99	0.288
Polyunsaturated fatty acids (PUFA)	White-backed	3.49	3.49	0.20	1.000
	Ash-grey	3.65 *	3.08	0.11	0.010
	Holstein	3.14	3.33	0.12	0.454
Omega 6	White-backed	2.33	2.39	0.13	0.848
	Ash-grey	2.88 *	2.32	0.10	0.007
	Holstein	2.62	2.81	0.09	0.362
Omega-3	White-backed	1.16	1.11	0.08	0.754
	Ash-grey	0.77	0.76	0.08	0.859
	Holstein	0.52	0.52	0.03	0989

Values are expressed as mean value ± SEM; Means in the same row followed by * are significantly different according to the Fisher LSD criterion test (*p* ≤ 0.05).

## Data Availability

The datasets used and/or analysed during the current study are available from the corresponding authors on reasonable request.
